# Modelling contaminant transport in fly ash–bentonite composite landfill liner: mechanism of different types of ions

**DOI:** 10.1038/s41598-020-68198-6

**Published:** 2020-07-09

**Authors:** Ankit Garg, Narala Gangadhara Reddy, He Huang, Poly Buragohain, Vinod Kushvaha

**Affiliations:** 1 Department of Civil and Environmental Engineering, Guangdong Engineering Center for Structure Safety and Health Monitoring, Shantou University, Shantou, China; 2 Department of Civil Engineering, Indian Institute of Technology Jammu, Jammu, India

**Keywords:** Hydrology, Civil engineering

## Abstract

Generated hazardous or toxic waste posses a serious threat if dumped into ponds or low lying areas which leads to contamination, this necessitates the effective landfill liner system. Mainly compacted clayey soils are used as an engineered barrier. Recently, composite materials have gained popularity as landfill liner materials, including the use of waste materials amended with low permeable soils. Though, studies on the composite optimum mix and its corresponding thickness are very scarce. Here, we evaluated the unconfined compressive strength and hydraulic conductivity of fly ash–bentonite composites. Efforts were also made to determine the thickness of landfill liner composite using a finite difference method (i.e. MATLAB). The results reveal that composite consists of 30% bentonite and 70% fly ash is suitable for landfill liner, which meets strength and permeability criteria. Numerical simulation for five major contaminants shows that the composite plays a crucial role in reducing the leaching of heavy metals and suggests an optimum thickness in the range of 126–154 cm. Overall, the findings of the study indicate that fly ash–bentonite composite can be used to solve real-life challenges in a sustainable way.

## Introduction

Landfill liner is a low-permeable barrier, which acts as a partition between the waste and its surrounding environment. Well constructed landfills are more secure than open dumping^1^. The fundamental factor influencing the nature of compacted clay liners is their low permeabilities, which should be as less as 1.0 × 10^–7^ cm/s suggested by RCRA (Subtitle D). To avoid contamination of groundwater (due to permeation of leachate), generally clayey soils are compacted to achieve desired permeability. Sand-bentonite composites are also used as engineered barriers or liners to prevent leaching of contaminants^2^. Other than hydraulic conductivity, strength also needs to be considered in assessing landfill liner material. Earlier studies and environmental guidelines proposed a minimum unconfined compressive strength (UCS) of 0.20 MPa to utilize as a landfill liner material^3^. Few studies reported that waste-bentonite composites satisfy the strength criteria when a correct mix proportion used^3,4^.

The utilization of wastes like fly ash solves the problems associated with waste management and also saves the extensive exploitation of natural materials^5,6^. Fly ash and bentonite can replace the sand-bentonite liners because of lack of available natural sand and an increase in the cost, which is widely used in other construction activities. Fly ash is known for its pozzolanic reactions, and that solidifies and gains strength over time when cured. Therefore, the curing period prompts an increase in strength and reduction of hydraulic conductivity. Recent studies have examined the amendment of fly ash to the soil, found the enhanced geotechnical properties such as cation exchange capacity, shear strength, and hydraulic conductivity^3,7,8^.

Problems in landfill lining system of a waste containment facilities can be escalated if built with unsuitable materials which accelerates the movement of solutes through desiccation cracks. Volatile organic chemicals (VOCs) or other organic solutes, which are the main contaminants because of their versatility and low concentrations at which they are lethal^9–11^. Therefore reactive materials that adsorb VOCs and decelerate their movement can make liners more effective. Fly ash is a potential material for the construction of landfill liner, which contains organic carbon, acts as a sorbent for VOCs^12^.

The significant hindrance is the point at which the heavy metals in leachate is more than the permissible limits, which is unsafe for drinking water. Researches have expressed that at a certain depth the concentration of contaminant in leachate increases with the increase of time^13,14^. The thickness of the liner is an essential deciding factor since it determines the strength and permeability. Till date, many researchers studied the use of analytical^15–19^ and numerical approaches^20^ for contamination transport (mainly organic) and to determine the liner thickness. However, the analytical methods are timing consuming and difficult for solving non-linear and differential equations^20^. On the other hand, studies related to numerical methods on fly ash–bentonite composite are scarce.

In the present study, investigations on the feasibility of fly ash–bentonite composites were explored. The objectives of this study are to (1) find the optimum dosage of fly ash and bentonite (composite) for its utilization in landfill liners; (2) determination of the corresponding thickness of landfill liner for selected fly ash–bentonite composite for landfill liner. The objectives were achieved by experimental and numerical model approach. A series of experimental tests were conducted to measure unconfined compressive strength and hydraulic conductivity of the fly ash–bentonite composites. Numerical simulations were performed on optimum dosage and thickness of the fly ash–bentonite composite to restrict leaching of five major contaminants.

## Materials and methods

### Materials and testing methodology

In the present study fly ash and bentonite were chosen to assess its use as a composite material for application in landfill liners. Fly ash was collected directly from the electrostatic precipitator of a power station located at Farakka, India and bentonite procured from a local vendor. The characterisation of both materials are conducted as per relevant standard codes and are presented in Table 1. As per ASTM standards the fly ash is classified as class F, and bentonite is classified as clay with high plasticity (i.e. CH). Buragohain et al.^3^ and Buragohain & Sreedeep^21^ have determined the specific surface area and cation exchange capacity of the same materials and these results are also summarized in Table 1. Both bentonite and fly ash majorly comprised of silica and aluminium oxides. The other chemical constituents are oxides of Fe, Ca, and Mg. The hydraulic conductivities for various combinations of fly ash–bentonite composites were conducted using the conventional falling head method. Unconfined compressive strength were determined as per the standard guidelines of ASTM for a curing period of 7, 14, and 28 days.

**Table 1:** Physical properties and chemical composition of materials.

Property	Fly ash	Bentonite	Chemical composition	Fly ash	Bentonite
Specific gravity (G)	2.07	2.82	SiO_2_	47.5	58.2
Particle size characteristics (%)			Al_2_O_3_	26.1	15.4
Fine sand (0.425–0.075 mm)	25	7	Fe_2_O_3_	8.4	3.3
Silt size (0.075–0.002 mm)	75	44	CaO	0.9	0.2
Clay size (0.02 mm)	0	49	MnO	0.2	1.3
Atterberg limits (%)			MgO	0.3	5.6
Liquid limit	Non-plastic	224	Loss on ignition	2.3	-
Plastic limit	–	31	Others	14.3	16
Plasticity index	–	193			
Classification	Class F	CH			
Specific surface area (m^2^/g)	1.4	219			
Cation exchange capacity (meq/100 g)*	1.23	56.9			
Sample minerals	Quartz, Mullite Magnetite	Quartz, Illite, Montmorillonite, Kaolinite			

*Buragohain et al.^3^.

### Numerical simulation

Leaching through compacted soil is controlled by an assortment of physical, chemical and organic procedures. The physical properties incorporate diffusion, advection, and dispersion^15–16^. The chemical process includes sorption, dissolution, complexation, hydrolysis/substitution and oxidation^17–19^. To predict the leaching of chlorides, zinc, iron, lead, and copper numerical parameters engaged with governing set of equations that depict the model processes should be accurately characterized. The one-dimensional vertical flow is numerically represented by the accompanying partial differential equation as expressed below and is the governing equation (Eq. 1)^15,19^.

1 $$ \frac{{\partial {\text{C}}_{{\text{t}}} }}{{\partial {\text{t}}}} = \frac{{{\text{D}}_{{\text{h}}} }}{{\text{R}}}\frac{{\partial^{2} {\text{C}}_{{\text{t}}} }}{{\partial^{2} {\text{x}}}} - \frac{{\text{v}}}{{{\theta R}}}\frac{{\partial {\text{C}}_{{\text{t}}} }}{{\partial {\text{x}}}} $$

C_t_ indicates the concentration corresponding to time, D_h_ is the hydrodynamic dispersion coefficient, v represents the seepage velocity, $\uptheta$ represents the volumetric water content, R indicates the retardation factor of the fly ash–bentonite layer (can be determined by the following the procedures given by Chen et al.^16^; Xie et al.^19^) and can be determined by Eq. (2).

2 $$ {\text{R}} = 1 + { }\frac{{{\text{K}}.{{ \rho }}_{{\text{d}}} }}{\upeta } $$

where ρ_d_ is the dry density; η is the porosity; K is the distribution coefficient of the fly ash- bentonite composite.

The initial condition of the liner system can then be expressed as (Chen et al.^16^; Xie et al.^9^; Feng et al.^18,19^):

3 $$ {\text{C}}_{{\text{t}}} \left( {x, 0} \right) = 0 $$

The bottom boundary of underlying soil is assumed to be a semi-infinite boundary (Xie et al.^9,10^):

4 $$ \frac{{\partial {\text{C}}_{{\text{t}}} \left( {\infty ,t} \right)}}{{\partial {\text{x}}}}{ } = { }0 $$

The Eq. (1) can be indicated by finite difference form using backward difference and Crank-Nicolson method as follows^22^

5 $$ \begin{aligned} \frac{{{\text{C}}_{{\text{i}}}^{{{\text{j}} + 1}} - {\text{C}}_{{\text{i}}}^{{\text{j}}} }}{{\Delta {\text{t}}}} & = \frac{{{\text{D}}_{{\text{h}}} }}{{2{\text{R}}}}\left( {\frac{{{\text{C}}_{{{\text{i}} + 1}}^{{{\text{j}} + 1}} - 2{\text{C}}_{{\text{i}}}^{{{\text{j}} + 1}} + {\text{C}}_{{{\text{i}} - 1}}^{{{\text{j}} + 1}} }}{{\left( {\Delta {\text{x}}} \right)^{2} }} + \frac{{{\text{C}}_{{{\text{i}} + 1}}^{{\text{j}}} - 2{\text{C}}_{{\text{i}}}^{{\text{j}}} + {\text{C}}_{{{\text{i}} - 1}}^{{\text{j}}} }}{{\left( {\Delta {\text{x}}} \right)^{2} }}} \right) \\ { } & \quad - \frac{{\text{v}}}{{2{\theta R}}}\left( {\frac{{{\text{C}}_{{{\text{i}} + 1}}^{{{\text{j}} + 1}} - 2{\text{C}}_{{\text{i}}}^{{{\text{j}} + 1}} + {\text{C}}_{{{\text{i}} - 1}}^{{{\text{j}} + 1}} }}{{\left( {\Delta {\text{x}}} \right)^{2} }}} \right) \\ \end{aligned} $$

This suggests

$$ {\text{C}}_{{\text{i}}}^{{{\text{j}} + 1}} - {\text{C}}_{{\text{i}}}^{{\text{j}}} = \frac{{{\text{D}}_{{\text{h}}} \Delta {\text{t}}}}{{2{\text{R}}\left( {\Delta {\text{x}}} \right)^{2} }}\left( {{\text{C}}_{{{\text{i}} + 1}}^{{{\text{j}} + 1}} - 2{\text{C}}_{{\text{i}}}^{{{\text{j}} + 1}} + {\text{C}}_{{{\text{i}} - 1}}^{{{\text{j}} + 1}} + {\text{C}}_{{{\text{i}} + 1}}^{{\text{j}}} - 2{\text{C}}_{{\text{i}}}^{{\text{j}}} + {\text{C}}_{{{\text{i}} - 1}}^{{\text{j}}} } \right) - \frac{{{\text{v}}\Delta {\text{t}}}}{{{\theta R}\left( {\Delta {\text{x}}} \right)^{2} }}\left( {{\text{C}}_{{{\text{i}} + 1}}^{{{\text{j}} + 1}} - 2{\text{C}}_{{\text{i}}}^{{{\text{j}} + 1}} + {\text{C}}_{{{\text{i}} - 1}}^{{{\text{j}} + 1}} } \right) $$

$$ {\text{Let}}\;{\text{A}} = { }\frac{{{\text{D}}_{{\text{h}}} \Delta {\text{t}}}}{{2{\text{R}}\left( {\Delta {\text{x}}} \right)^{2} }}\;{\text{and}}\;{\text{B}} = { }\frac{{{\text{v}}\Delta {\text{t}}}}{{2{\theta R}\left( {\Delta {\text{x}}} \right)^{2} }} $$

6 $$ {\text{C}}_{{\text{i}}}^{{{\text{j}} + 1}} - {\text{C}}_{{\text{i}}}^{{\text{j}}} = {\text{A}}\left( {{\text{C}}_{{{\text{i}} + 1}}^{{{\text{j}} + 1}} - 2{\text{C}}_{{\text{i}}}^{{{\text{j}} + 1}} + {\text{C}}_{{{\text{i}} - 1}}^{{{\text{j}} + 1}} + {\text{C}}_{{{\text{i}} + 1}}^{{\text{j}}} - 2{\text{C}}_{{\text{i}}}^{{\text{j}}} + {\text{C}}_{{{\text{i}} - 1}}^{{\text{j}}} } \right) - {\text{B}}\left( {{\text{C}}_{{{\text{i}} + 1}}^{{{\text{j}} + 1}} - 2{\text{C}}_{{\text{i}}}^{{{\text{j}} + 1}} + {\text{C}}_{{{\text{i}} - 1}}^{{{\text{j}} + 1}} } \right) $$

Implies,

7 $$ {\text{C}}_{{\text{i}}}^{{{\text{j}} + 1}} = \frac{{{\text{C}}_{{\text{i}}}^{{\text{j}}} \left( {1 - 2{\text{A}}} \right) + \left( {{\text{C}}_{{{\text{i}} + 1}}^{{{\text{j}} + 1}} - {\text{C}}_{{{\text{i}} - 1}}^{{{\text{j}} + 1}} } \right)\left( {{\text{A}} - {\text{B}}} \right) + {\text{A}}\left( {{\text{C}}_{{{\text{i}} + 1}}^{{\text{j}}} + {\text{C}}_{{{\text{i}} - 1}}^{{\text{j}}} } \right)}}{{1 + 2{\text{A}} + 2{\text{B}}}} $$

The value of $\Delta {\text{t}}$ and $\Delta {\text{x}}$ have been picked such that the following suitability equation is fulfilled. Equation (7), which is a discrete form of Eq. (1) has been coded in MATLAB for running the simulation.

$$ \frac{{{\text{D}}_{{\text{h}}} \Delta {\text{t}}}}{{2{\text{R}}\left( {\Delta {\text{x}}} \right)^{2} }} + \frac{{{\text{v}}\Delta {\text{t}}}}{{2{\theta R}\left( {\Delta {\text{x}}} \right)^{2} }} \le \frac{1}{2} $$

Table 2 summarizes the parameters used in the study. The concentrations of contaminants are taken based on the available literature, which are reported at various landfill sites^12,23,24^. The corresponding allowable levels suggested by WHO are also represented in Table 2. The above conditions were used in the numerical difference model for a simulation period of up to 100 years.

**Table. 2:** Parameters and concentration of leachates used for simulation.

Parameters	Value	Contaminant	Concentration (ppm)	Permissible Concentration (ppm)
Seepage velocity (cm/year)	0.2346	Chloride	4,000	250
$\Delta t$ (year)	1	Zinc	3.2	3
$\Delta x$ (cm)	14	Iron	73.6	3
$\theta$	0.33	Lead	19.4	0.05
		Copper	62.6	2

## Results and discussion

### Determination of optimum fly ash–bentonite composite

**Figure 1:**
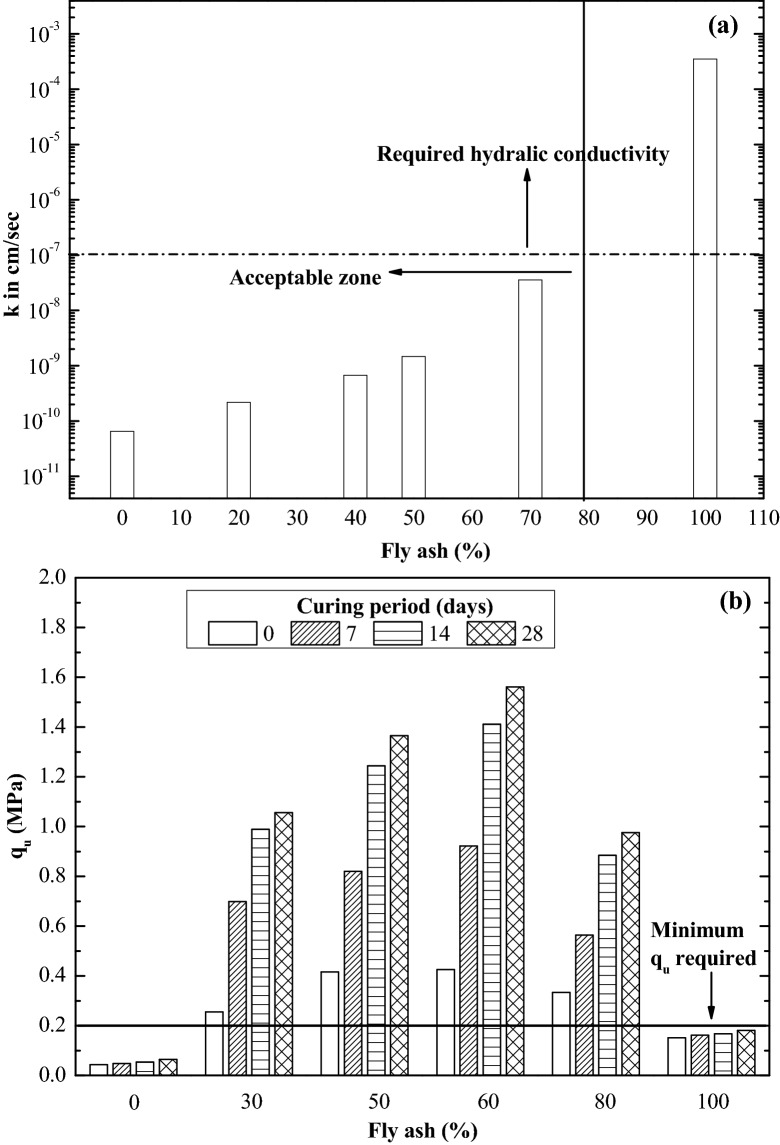
(**a**) Variation of hydraulic conductivity with percentage of fly ash and (**b**) unconfined compressive strength with percentage of fly ash.

UCS and hydraulic conductivity of the fly ash–bentonite composite were investigated to determine their optimum composite for landfill liner. The hydraulic conductivity of fly ash–bentonite composite is plotted, taking bentonite percentage into account, as shown in Fig. 1a. The hydraulic conductivity of fly ash alone was 3.55 × 10^–4^ cm/s (comparable to that of fine sand), such high hydraulic conductivity excludes its direct utilisation for landfill liner applications^7^. However, the hydraulic conductivity of fly ash reduced with a percentage increase of bentonite. The reduction is roughly four orders of magnitude with 30% bentonite addition. Further addition of bentonite, decreased hydraulic conductivity significantly compared to raw fly ash. The decline is mainly due to the smaller particle sizes of bentonite, which acts as pore filler in fly ash. It can be observed from Fig. 1a composite having a 30% bentonite and 70% fly ash satisfies the hydraulic conductivity criteria^3^.

Fly ash is known as a pozzolanic material; the chemical reactions take plays over time and results in improvement of strength. Thus it is inferred that the strength of the composite is influenced by the curing period. The UCS test performed on various composites for a curing period of 7, 14, and 28 days are plotted in bar chart form. As depicted in Fig. 1b it can be observed that the increase in curing period improves the strength of composites. A composite having 60% fly ash and 40% bentonite shows the maximum UCS and also satisfies the minimum UCS criteria (> 0.2 MPa) proposed by USEPA^25^ and Buragohain et al.^3^. Though 60% bentonite has high UCS, keeping in mind that, the use more fly ash and to minimise the usage of bentonite for a sustainable solution a composite with 30% bentonite and 70% fly ash is recommended for practical applications and to utilize maximum waste, i.e. fly ash. To estimate the thickness of the landfill liner composite with 30% bentonite and 70% fly ash was used for the numerical modelling.

### Contamination transport and thickness of landfill liner

**Figure 2:**
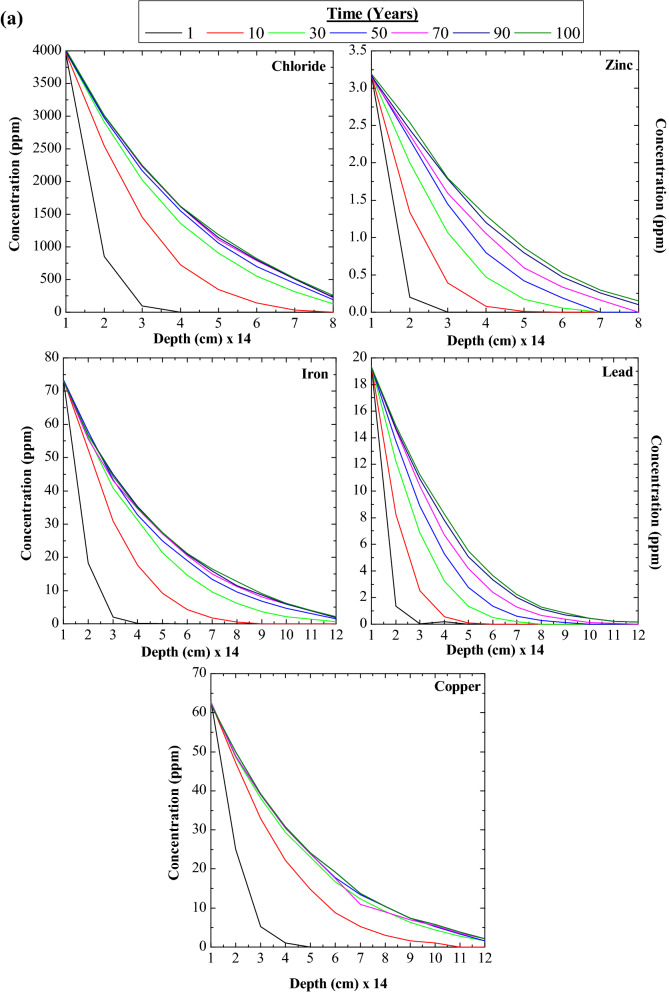

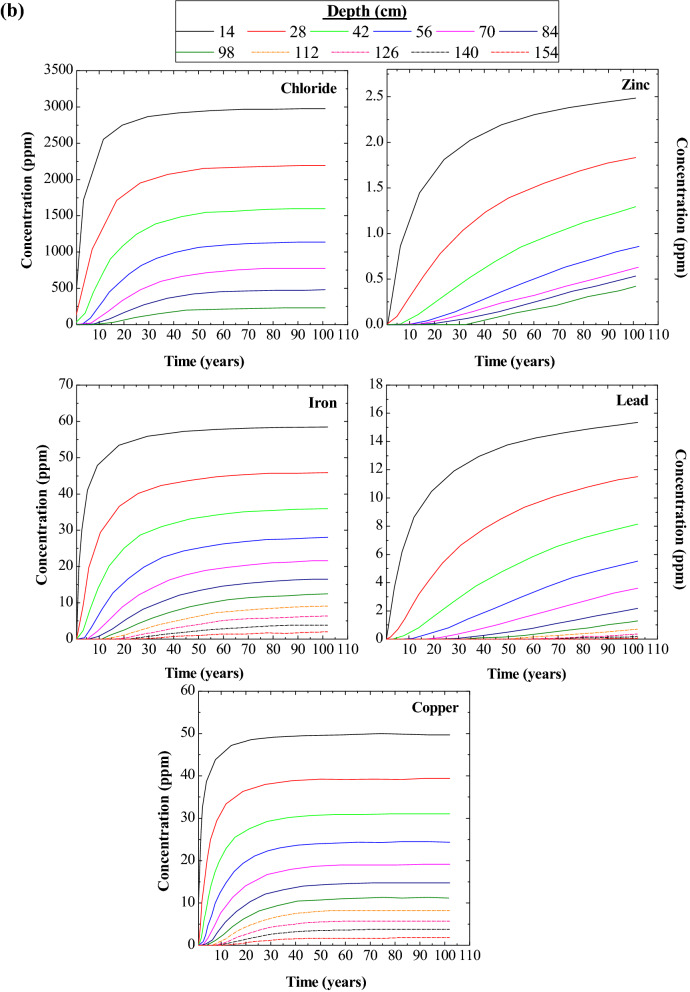
(**a**)Variation of leachate concentrations with depth. (**b**) Variation of leachate concentration with time.

Five ionic concentration and their maximum permissible limits are given in Table 2. The hydromatic dispersion and retardation factors were chosen as per the studies of Salami et al.^22^, Chalermyanont et al.^12^, Jhamnani and Singh^23^, and Aswathi et al.^24^. It can be seen from Fig. 2a that as the depth increases the concentration of the contaminant leaching into the landfill also decreased for all the periods. However with the increase in time the chlorides, iron, and copper are stabilising after a time interval of 50 to 70 years (refer Fig. 2b) but, lead, and zinc are not stabilised compared to other three leachates. Such divergence of values shows the low reactivity of zinc and lead towards fly ash. In any case, considering the landfill to last for 100 years, the concentration of zinc to which it can increase is tolerable (ref Fig. 2b). Also, Fig. 2a shows no convergence of concentration with respect to depth, which presumes that the considered depth scale does not resist seepage completely. Overall the numerical model suggests a min of 126 cm thickness for chlorides, iron, and copper and 154 cm for lead and zinc for a design period of 100 years. The composite which has a permeability of less than or equal to 1.0 × 10^–7^ cm/s has a seepage movement of 30 cm for 30 years of period^26^. This observation validates the thickness of landfill liner estimated with the help of a numerical model.

## Conclusions

The study analysed the use of fly ash–bentonite composite as a landfill liner material. The unconfined compressive strength and hydraulic conductivity of composites were evaluated critically. Further quantified the optimum percentage mix for composite using design criteria and estimated is the corresponding thickness for landfill liner using numerical model simulations. Based on the study, it was found that 70% fly ash and 30% bentonite composite can be used as an optimum mix for landfill liners application which is meeting the strength (> 0.2 MPa) and permeability (1.0 × 10^–7^ cm/s) criteria. A series of numerical model studies suggest a thickness of 126 to 154 cm as a landfill liner for a design period of 100 years. The recommended landfill liner thickness shows the reduction of leachates passing through the composite liner into the groundwater. Overall, the findings of the study indicate that fly ash–bentonite composites have good potential for application. The composites not only solve the prevention of groundwater contamination and also fly ash can be utilised beneficially in a sustainable way.

The conclusions are mainly from the laboratory and numerical model studies. Studies further can be extended considering the organic contaminants and analytical methods. Wetting–drying cycles on fly ash–bentonite composite are also essential to understand the long term behaviour, including cracking. Future studies, especially the weakening effect of real landfill leachate to the composite, should be estimated for practical applications.
